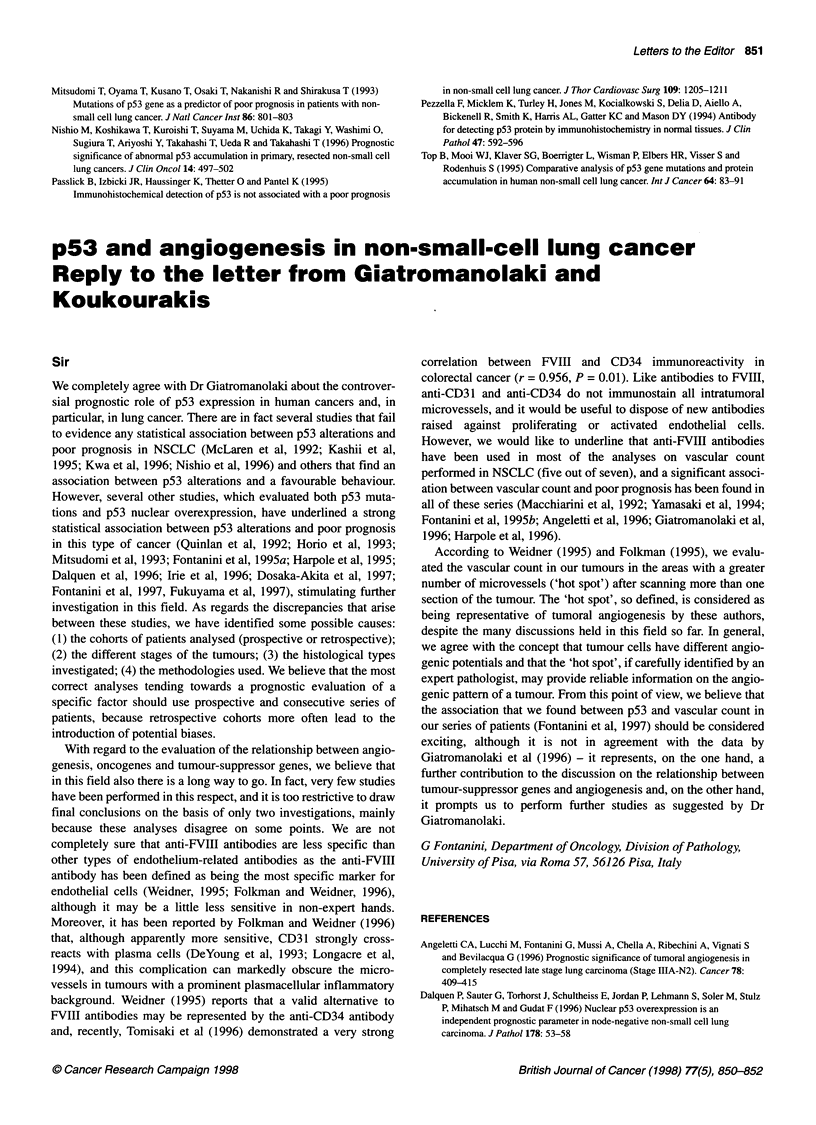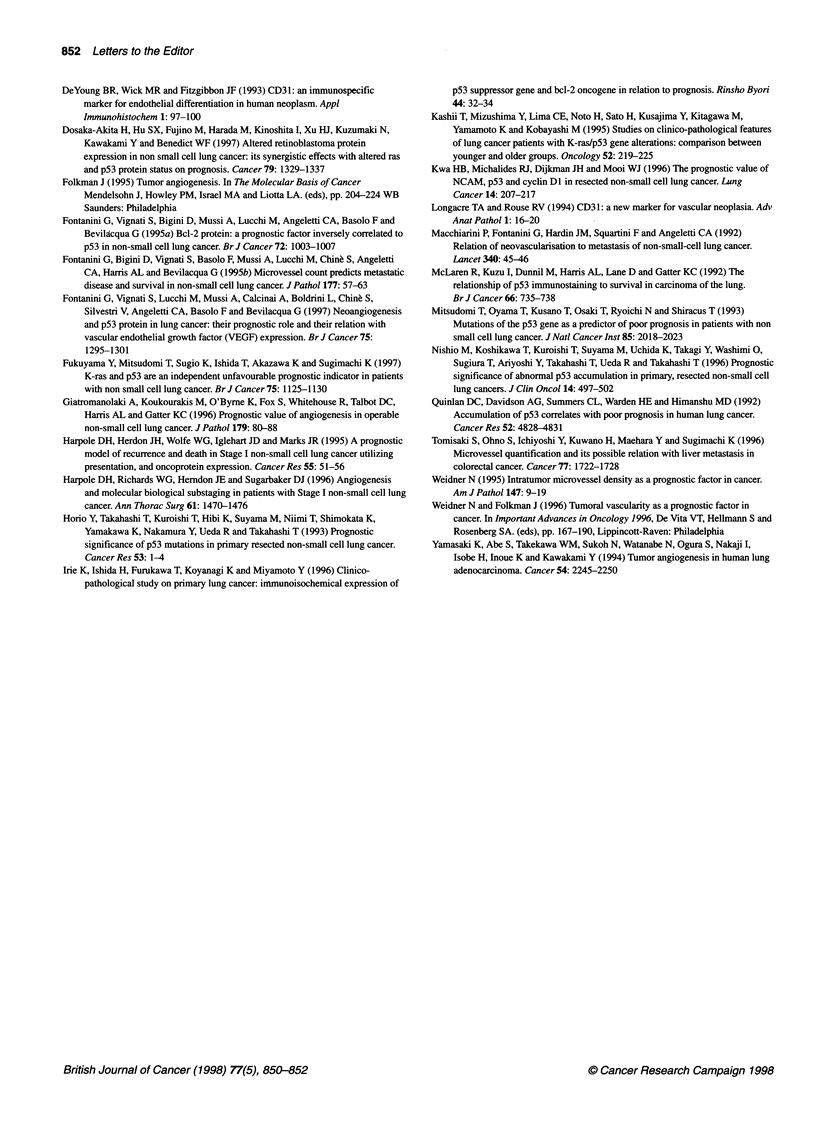# p53 and angiogenesis in non-small-cell lung cancer

**Published:** 1998-03

**Authors:** G Fontanini


Nishio M, Koshikawa T, Kuroishi T, Suyama M, Uchida K, Takagi Y, Washimi O, Sugiura T, Ariyoshi Y, Takahashi T, Ueda R and Takahashi T (1996) Prognostic significance of abnormal p53 accumulation in primary, resected non-small cell lung cancers. *J Clin Oncol* **14**: 497–502

### p53 and angiogenesis in non-small-cell lung cancer Reply to the letter from Giatromanolaki and Koukourakis

### Sir

We completely agree with Dr Giatromanolaki about the controversial prognostic role of p53 expression in human cancers and, in particular, in lung cancer. There are in fact several studies that fail to evidence any statistical association between p53 alterations and poor prognosis in NSCLC (McLaren et al, 1992; Kashii et al, 1995; Kwa et al, 1996; Nishio et al, 1996) and others that find an association between p53 alterations and a favourable behaviour. However, several other studies, which evaluated both p53 mutations and p53 nuclear overexpression, have underlined a strong statistical association between p53 alterations and poor prognosis in this type of cancer (Quinlan et al, 1992; Horio et al, 1993; Mitsudomi et al, 1993; Fontanini et al, 1995*a*; Harpole et al, 1995; Dalquen et al, 1996; Irie et al, 1996; Dosaka-Akita et al, 1997; Fontanini et al, 1997, Fukuyama et al, 1997), stimulating further investigation in this field. As regards the discrepancies that arise between these studies, we have identified some possible causes: (1) the cohorts of patients analysed (prospective or retrospective); (2) the different stages of the tumours; (3) the histological types investigated; (4) the methodologies used. We believe that the most correct analyses tending towards a prognostic evaluation of a specific factor should use prospective and consecutive series of patients, because retrospective cohorts more often lead to the introduction of potential biases.

With regard to the evaluation of the relationship between angiogenesis, oncogenes and tumour-suppressor genes, we believe that in this field also there is a long way to go. In fact, very few studies have been performed in this respect, and it is too restrictive to draw final conclusions on the basis of only two investigations, mainly because these analyses disagree on some points. We are not completely sure that anti-FVIII antibodies are less specific than other types of endothelium-related antibodies as the anti-FVIII antibody has been defined as being the most specific marker for endothelial cells (Weidner, 1995; Folkman and Weidner, 1996), although it may be a little less sensitive in non-expert hands. Moreover, it has been reported by Folkman and Weidner (1996) that, although apparently more sensitive, CD31 strongly cross-reacts with plasma cells (DeYoung et al, 1993; Longacre et al, 1994), and this complication can markedly obscure the microvessels in tumours with a prominent plasmacellular inflammatory background. Weidner (1995) reports that a valid alternative to FVIII antibodies may be represented by the anti-CD34 antibody and, recently, Tomisaki et al (1996) demonstrated a very strong correlation between FVIII and CD34 immunoreactivity in colorectal cancer ($r = 0.956$, $P = 0.01$). Like antibodies to FVIII, anti-CD31 and anti-CD34 do not immunostain all intratumoral microvessels, and it would be useful to dispose of new antibodies raised against proliferating or activated endothelial cells. However, we would like to underline that anti-FVIII antibodies have been used in most of the analyses on vascular count performed in NSCLC (five out of seven), and a significant association between vascular count and poor prognosis has been found in all of these series (Macchiarini et al, 1992; Yamasaki et al, 1994; Fontanini et al, 1995*b*; Angeletti et al, 1996; Giatromanolaki et al, 1996; Harpole et al, 1996).

According to Weidner (1995) and Folkman (1995), we evaluated the vascular count in our tumours in the areas with a greater number of microvessels ('hot spot') after scanning more than one section of the tumour. The 'hot spot', so defined, is considered as being representative of tumoral angiogenesis by these authors, despite the many discussions held in this field so far. In general, we agree with the concept that tumour cells have different angiogenic potentials and that the 'hot spot', if carefully identified by an expert pathologist, may provide reliable information on the angiogenic pattern of a tumour. From this point of view, we believe that the association that we found between p53 and vascular count in our series of patients (Fontanini et al, 1997) should be considered exciting, although it is not in agreement with the data by Giatromanolaki et al (1996) – it represents, on the one hand, a further contribution to the discussion on the relationship between tumour-suppressor genes and angiogenesis and, on the other hand, it prompts us to perform further studies as suggested by Dr Giatromanolaki.

*G Fontanini, Department of Oncology, Division of Pathology, University of Pisa, via Roma 57, 56126 Pisa, Italy*

### REFERENCES

Angeletti CA, Lucchi M, Fontanini G, Mussi A, Chella A, Ribechini A, Vignati S and Bevilacqua G (1996) Prognostic significance of tumoral angiogenesis in completely resected late stage lung carcinoma (Stage IIIA-N2). *Cancer* **78**: 409–415

Dalquen P, Sauter G, Torhorst J, Schultheiss E, Jordan P, Lehmann S, Soler M, Stulz P, Mihatsch M and Gudat F (1996) Nuclear p53 overexpression is an independent prognostic parameter in node-negative non-small cell lung carcinoma. *J Pathol* **178**: 53–58

© Cancer Research Campaign 1998

DeYoung BR, Wick MR and Fitzgibbon JF (1993) CD31: an immunospecific marker for endothelial differentiation in human neoplasm. *Appl Immunohistochem* **1**: 97–100

Dosaka-Akita H, Hu SX, Fujino M, Harada M, Kinoshita I, Xu HJ, Kuzumaki N, Kawakami Y and Benedict WF (1997) Altered retinoblastoma protein expression in non small cell lung cancer: its synergistic effects with altered ras and p53 protein status on prognosis. *Cancer* **79**: 1329–1337

Folkman J (1995) Tumor angiogenesis. In *The Molecular Basis of Cancer* Mendelsohn J, Howley PM, Israel MA and Liotta LA. (eds), pp. 204–224 WB Saunders: Philadelphia

Fontanini G, Vignati S, Bigini D, Mussi A, Lucchi M, Angeletti CA, Basolo F and Bevilácqua G (1995*a*) Bcl-2 protein: a prognostic factor inversely correlated to p53 in non-small cell lung cancer. *Br J Cancer* **72**: 1003–1007

Fontanini G, Bigini D, Vignati S, Basolo F, Mussi A, Lucchi M, Chinè S, Angeletti CA, Harris AL and Bevilacqua G (1995*b*) Microvessel count predicts metastatic disease and survival in non-small cell lung cancer. *J Pathol* **177**: 57–63

Fontanini G, Vignati S, Lucchi M, Mussi A, Calcinai A, Boldrini L, Chinè S, Silvestri V, Angeletti CA, Basolo F and Bevilacqua G (1997) Neoangiogenesis and p53 protein in lung cancer: their prognostic role and their relation with vascular endothelial growth factor (VEGF) expression. *Br J Cancer* **75**: 1295–1301

Fukuyama Y, Mitsudomi T, Sugio K, Ishida T, Akazawa K and Sugimachi K (1997) K-ras and p53 are an independent unfavourable prognostic indicator in patients with non small cell lung cancer. *Br J Cancer* **75**: 1125–1130

Giatromanolaki A, Koukourakis M, O'Byrne K, Fox S, Whitehouse R, Talbot DC, Harris AL and Gatter KC (1996) Prognostic value of angiogenesis in operable non-small cell lung cancer. *J Pathol* **179**: 80–88

Harpole DH, Herdon JH, Wolfe WG, Iglehart JD and Marks JR (1995) A prognostic model of recurrence and death in Stage I non-small cell lung cancer utilizing presentation, and oncoprotein expression. *Cancer Res* **55**: 51–56

Harpole DH, Richards WG, Herndon JE and Sugarbaker DJ (1996) Angiogenesis and molecular biological substaging in patients with Stage I non-small cell lung cancer. *Ann Thorac Surg* **61**: 1470–1476

Horio Y, Takahashi T, Kuroishi T, Hibi K, Suyama M, Niimi T, Shimokata K, Yamakawa K, Nakamura Y, Ueda R and Takahashi T (1993) Prognostic significance of p53 mutations in primary resected non-small cell lung cancer. *Cancer Res* **53**: 1–4

Irie K, Ishida H, Furukawa T, Koyanagi K and Miyamoto Y (1996) Clinico-pathological study on primary lung cancer: immunoisochemical expression of p53 suppressor gene and bcl-2 oncogene in relation to prognosis. *Rinsho Byori* **44**: 32–34

Kashii T, Mizushima Y, Lima CE, Noto H, Sato H, Kusajima Y, Kitagawa M, Yamamoto K and Kobayashi M (1995) Studies on clinico-pathological features of lung cancer patients with K-ras/p53 gene alterations: comparison between younger and older groups. *Oncology* **52**: 219–225

Kwa HB, Michalides RJ, Dijkman JH and Mooi WJ (1996) The prognostic value of NCAM, p53 and cyclin D1 in resected non-small cell lung cancer. *Lung Cancer* **14**: 207–217

Longacre TA and Rouse RV (1994) CD31: a new marker for vascular neoplasia. *Adv Anat Pathol* **1**: 16–20

Macchiarini P, Fontanini G, Hardin JM, Squartini F and Angeletti CA (1992) Relation of neovascularisation to metastasis of non-small-cell lung cancer. *Lancet* **340**: 45–46

McLaren R, Kuzu I, Dunnil M, Harris AL, Lane D and Gatter KC (1992) The relationship of p53 immunostaining to survival in carcinoma of the lung. *Br J Cancer* **66**: 735–738

Mitsudomi T, Oyama T, Kusano T, Osaki T, Ryoichi N and Shiracus T (1993) Mutations of the p53 gene as a predictor of poor prognosis in patients with non small cell lung cancer. *J Natl Cancer Inst* **85**: 2018–2023

Nishio M, Koshikawa T, Kuroishi T, Suyama M, Uchida K, Takagi Y, Washimi O, Sugiura T, Ariyoshi Y, Takahashi T, Ueda R and Takahashi T (1996) Prognostic significance of abnormal p53 accumulation in primary, resected non-small cell lung cancers. *J Clin Oncol* **14**: 497–502

Quinlan DC, Davidson AG, Summers CL, Warden HE and Himanshu MD (1992) Accumulation of p53 correlates with poor prognosis in human lung cancer. *Cancer Res* **52**: 4828–4831

Tomisaki S, Ohno S, Ichiyoshi Y, Kuwano H, Maehara Y and Sugimachi K (1996) Microvessel quantification and its possible relation with liver metastasis in colorectal cancer. *Cancer* **77**: 1722–1728

Weidner N (1995) Intratumor microvessel density as a prognostic factor in cancer. *Am J Pathol* **147**: 9–19

Weidner N and Folkman J (1996) Tumoral vascularity as a prognostic factor in cancer. In *Important Advances in Oncology 1996*, De Vita VT, Hellmann S and Rosenberg SA. (eds), pp. 167–190, Lippincott-Raven: Philadelphia

Yamasaki K, Abe S, Takekawa WM, Sukoh N, Watanabe N, Ogura S, Nakaji I, Isobe H, Inoue K and Kawakami Y (1994) Tumor angiogenesis in human lung adenocarcinoma. *Cancer* **54**: 2245–2250

*© Cancer Research Campaign 1998*